# Determinants of the lost to follow-up status among patients with tuberculosis who emigrated to the Republic of Korea: a mixed-method study

**DOI:** 10.3389/fpubh.2025.1641182

**Published:** 2025-09-12

**Authors:** Sumin Jeon, Ji Yeon Lee, Ina Jeong, Sooim Sin, Inhan Lee, Younghyun Kim, Ah Yeon Han, Seung-Eun Lee, Soonryu Seo, Hyungjun Kim, Yunhyung Kwon, Chieeun Song, Joon-Sung Joh, Sung Hye Kim

**Affiliations:** ^1^Graduate School of Public Health, Hanyang University, Seoul, Republic of Korea; ^2^Division of Pulmonary and Critical Care Medicine, Department of Internal Medicine, National Medical Center, Seoul, Republic of Korea; ^3^Division of Tuberculosis Prevention and Control, Korea Disease Control and Prevention Agency, Cheongju, Republic of Korea; ^4^Center for Global Health Practice, Institute of Health and Society, Hanyang University College of Medicine, Seoul, Republic of Korea

**Keywords:** tuberculosis, lost to follow-up, migrants, Republic of Korea, private-public mix

## Abstract

**Introduction:**

Tuberculosis (TB) remains a significant global health concern, with foreign migrants in the Republic of Korea (ROK) representing a particularly vulnerable group; despite comprising only 3.5% of the population, they account for over 5% of annual TB cases and exhibit disproportionately high rates of lost to follow up (LTFU) from treatment compared to native Koreans. This mixed-methods study aimed to identify key factors influencing non-adherence to treatment and LTFU among migrants.

**Methods:**

Utilizing national TB surveillance data from 2016 to 2018 for 4,011 migrant and 64,620 native patients, quantitative analysis were employed to identify factors associated with LTFU for migrants. Complementary in-depth qualitative interviews with Public-Private Mix (PPM) nurses provided deeper insights into barriers to adherence.

**Results:**

The study revealed a significantly higher LTFU rate (21.5%) among migrant patients compared to domestic patients (2.3%). Key contributing factors included nationality (highest crude odds for migrants from Thailand, Central, and North Asia), living arrangements (increased risk for those not with family or living alone), and male gender. Drug-resistant TB made patients over four times more likely to discontinue treatment and systemic issues such as frequent care transfers and the presence of comorbidities. Qualitative findings highlighted inadequate patient education and misconceptions about TB severity (often seen as a “mild cold”), leading to premature discontinuation. Poor medical interpretation services and low awareness among migrants of free TB treatment under the PPM program were also critical barriers.

**Discussion:**

These findings imply that high LTFU among migrant patients is multifactorial, stemming from personal, clinical, and systemic issues. Addressing this disparity requires targeted interventions, including culturally tailored multilingual educational campaigns, improved medical interpretation, and increased awareness of PPM program eligibility and free treatment. Streamlining interfacility care transfer processes (such as the “Tuberculosis Relief Belt” initiative), expanding PPM coverage, and ensuring access to specialized care for comorbid conditions are also essential. Addressing these multifaceted challenges is critical to reducing LTFU rates and enhancing treatment continuity and outcomes, thereby advancing TB control efforts in ROK’s shifting migration context.

## 1 Introduction

Tuberculosis (TB) remains a significant global health challenge (1). While the Republic of Korea (ROK) has drastically reduced the incidence from 5,168 cases per 100,000 in 1965 to 38 cases per 100,000 in 2023 (2, 3) and TB-related mortality has also shown marked improvement, critical public health challenges remain (3–5). TB is designated as a nationally notifiable disease under the Infectious Diseases Control and Prevention Act, mandating that all new diagnoses be reported to community health centers within 24 h (6). Established in 2000, the Korean National Tuberculosis Surveillance System (KNTSS) continuously monitors patient demographics, diagnostic outcomes, treatment modalities, and overall patient prognoses. Furthermore, the introduction of the Public-Private Mix (PPM) program in 2011, which emphasizes nurse-led patient monitoring, contributed to a substantial 43.2% reduction in TB-related deaths from 2013 to 2023 (5, 7).

Migrant communities represent particularly vulnerable groups in the ROK’s TB landscape. Although foreign-born individuals account for only 3.5% of the total population (8), they now contribute to more than 5% of annual TB cases, a significant increase from 0.3% in 2001 to 5.7% in 2023 (9, 10). Notably, Chinese nationals, especially Korean Chinese from northeastern China, account for nearly half of all TB cases among migrants (11, 12). Although the general population in the ROK experienced a decline in LTFU—from 5.3% in 2011 to 2.4% in 2018 (13, 14)—migrant populations continue to face disproportionately high risks (12). Interestingly, although migrants had lower overall odds of treatment success (odds ratio [OR] 0.71, 95% confidence interval [CI] 0.46–1.12), this difference was not statistically significant. However, migrants demonstrate lower rates of treatment completion, as verified through microbiological confirmation, suggesting that despite effective initial therapy, disengagement often occurs during the continuation phase (12).

Migrant patients with TB face multifaceted barriers to continuing treatment (15). Socioeconomic challenges—including financial constraints, unstable employment, and housing insecurity—often intersect with healthcare system obstacles such as limited access to specialized PPM nurses (16–18). In addition, clinical factors such as drug-resistant TB and comorbid conditions such as diabetes and cancer further increase the risk of LTFU (12, 19–22). Despite the growing recognition of these challenges, the specific determinants of LTFU among migrant populations in the ROK remain underexplored. This study addresses this gap by analyzing TB cases among migrants reported to the KNTSS between 2016 and 2018 and comparing them with native TB cases to identify key risk factors for treatment discontinuation. Complementary in-depth interviews with PPM nurses working in regions with large migrant populations provided additional insights into the unique barriers faced by these patients.

## 2 Materials and methods

This mixed method study was designed to investigate the factors affecting non-adherence and LTFU from TB treatment among foreign migrants in ROK, comparing them to native Korean TB patients. The study combined a retrospective cohort analysis of national health system data with complementary qualitative interviews.

### 2.1 Study design and setting

The quantitative element involved a retrospective cohort study utilizing national TB surveillance data from ROK, for the period between January 2016 and December 2018. The qualitative component involved in-depth interviews with Public-Private Mix (PPM) nurses.

### 2.2 Participants and data source

**Figure 1:**
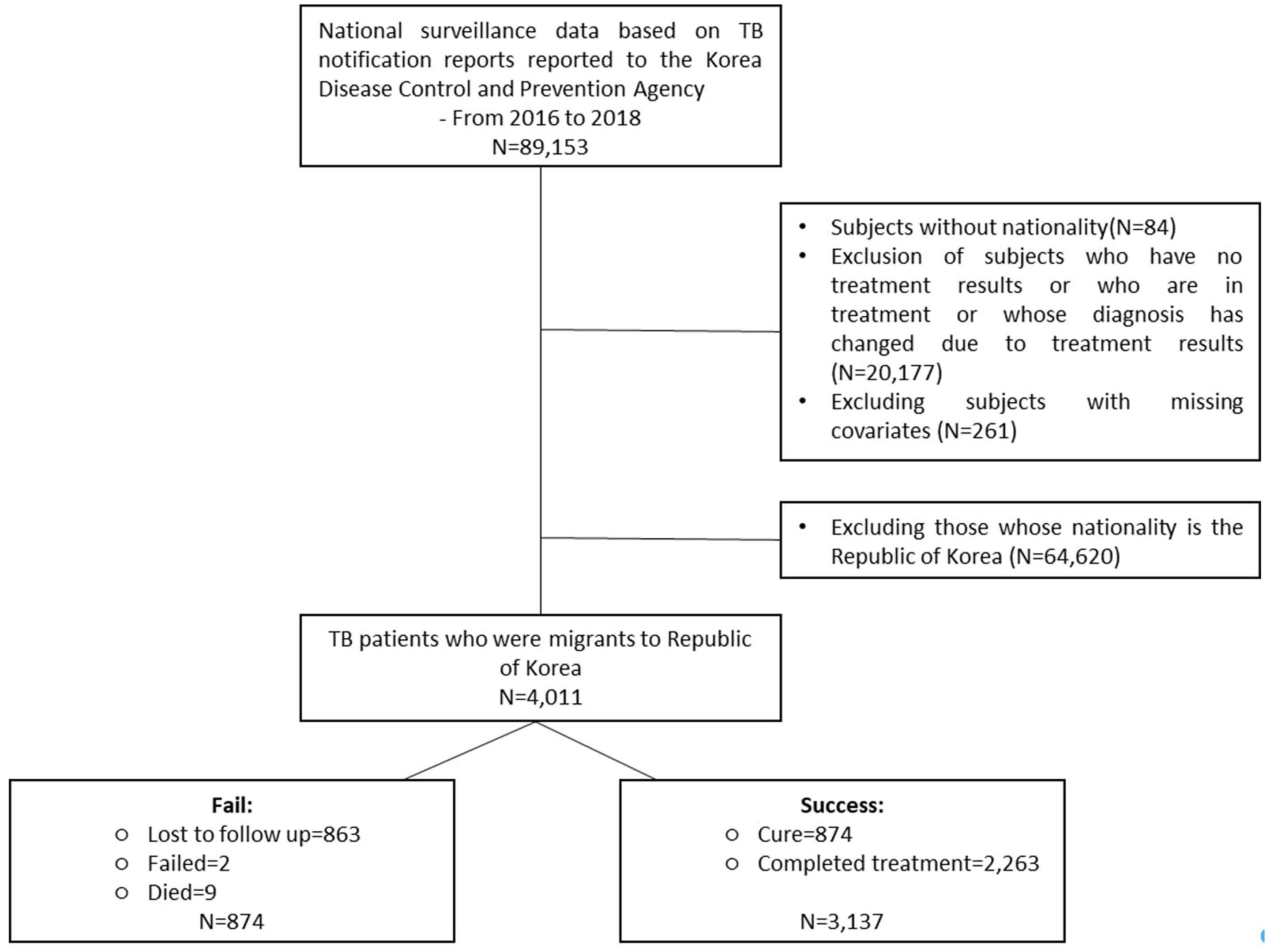
Flow diagram of data management.

The initial dataset comprised of 89,153 notified TB cases. After exclusions for diagnostic changes, ongoing treatment, missing covariates or unrecorded nationality, the final study cohort comprised 64,620 Korean and 4,011 foreign nationals (Figure 1). Participants for qualitative interviews included specialist doctors and PPM nurses from various hospital settings (Supplementary file S1), selected through a purposive sampling approach.

### 2.3 Operational definition and variables

Thirteen variables were examined and organized into three domains:

- Sociodemographic profiles: This included sex, age, nationality, living arrangement, smoking status, and geographical region of residence.
- Disease profiles and health system: This included TB treatment history, disease site, drug resistance status, PPM program enrollment, care transfer, and type of treatment facility.
- Comorbidities: This domain assessed the presence of diabetes, cancer, or other chronic conditions.

TB case definitions, recommended treatment regimens and monitoring framework, and reporting procedures are summarized in the Supplementary file S2, as outlined in the National TB Management Guideline (23). Treatment outcomes were defined according to the 2020 World Health Organization (WHO) guidelines (23), classifying cases as either “successful” (cure or treatment completion), or “unsuccessful” (treatment failure, LFTU, or death). The primary outcome of interest was LTFU, which was defined as a treatment interruption lasting 2 months or more.

### 2.4 Data collection

Quantitative data were systematically collected from the Korean National Tuberculosis Surveillance System (KNTSS), which maintains comprehensive records on patient demographics, diagnostic outcomes, treatment modalities, and prognoses. For the qualitative phase, in-person, individual interviews were conducted with PPM nurses at their respective institutions, lasting up to an hour and audio-recorded with consent. All personal identifiers were removed during verbatim transcription to ensure confidentiality. No incentives were provided for participation.

### 2.5 Data analysis

For quantitative analysis, chi-square tests and multivariate logistic regression analyses were employed to identify risk factors associated with LTFU. Data were analyzed using STATA 17. Qualitative data were analyzed using a thematic approach, with themes emerging inductively from the transcripts. Researchers identified recurring patterns and salient issues, which were then synthesized into three principal themes: Personal Factors (patient attitudes, behaviors, and circumstances), Structural Factors (living arrangements, socioeconomic and environmental barriers), and Health Service-Related Factors (resource limitations, coordination gaps, and systemic challenges). This inductive method provided critical context for the quantitative findings and illuminated specific barriers contributing to treatment discontinuation among migrant TB patients. The consistency of these themes was regularly reviewed throughout the interview process.

## 3 Results

### 3.1 Characteristics of patients with TB: migrants versus residents

This retrospective cohort study analyzed TB cases in ROK between 2016 and 2018, including 4,011 migrant patients, 2,415 males and 1,596 females and 64,620 nationals. Participants’ demographic characteristics are presented in Supplementary file S3. Registered foreigners increased from 1,161,677 in 2016 to 246,626 in 2018, with TB incidence among migrants declining over time (221.1 to 144.5 per 100,000), averaging 178.2, approximately 2.5 times the national incidence (71.0 per 100,000) (8, 10, 24, 25). Nationality-specific disparities were evident: Mongolian nationals had the highest TB incidence (216.7), while Uzbek nationals had the lowest (48.5).

Migrant patients were predominantly aged 30–49 and more likely to live alone (54.4% versus. 45.0%). They had a higher prevalence of drug-resistant TB (3.8% vs. 1.5%, *p* < 0.001) and lower access to PPM nurses (52.9% vs. 73.9%). Although comorbidities, such as diabetes (4.2% vs. 14.1%) and cancer (2.0% vs. 6.4%), were less common, their presence had a more pronounced impact on treatment discontinuation. Migrants experienced significantly higher LTFU rates (21.5% vs. 2.3%, *p* < 0.001) and lower treatment completion (56.4% vs. 75.3%), despite similar cure rates (21.8% vs. 20.1%).

### 3.2 Sex-based differences among migrants

Female migrants were concentrated in the ≤30 and ≥70 age groups, while males were predominantly aged 30–69. Men were more likely to live alone (59.5%) and women were more likely to live with family (47.8%). Smoking patterns differed notably—95.3% of women were non-smokers versus 55.2% of men who were current or former smokers.

### 3.3 Determinants of lost to follow-up among migrants

Unadjusted analysis identified several key risk factors for LTFU, including Thai origin (OR 5.28), Central and North Asian nationality (OR 2.96), living alone (OR 2.39), drug-resistant TB (OR, 3.78), care transfers (OR, 7.10) diabetes (OR 1.77) and cancer (OR 1.74), and receiving care in clinics or hospitals as opposed to general hospitals (OR range: 1.51–1.90) (Table 1). Female sex was associated with lower odds of LTFU (OR, 0.73).

**Table 1:** Univariable analysis of lost to follow-up among TB patients who were migrants, registered and treated from 2016 to 2018.

Variables		Crude OR	95% CI		*p*-value
			Lower	Upper	
Yearly distribution					
Year	2016	1			
	2017	0.94	0.78	1.12	0.473
	2018	0.95	0.79	1.14	0.573
Sociodemographic profile					
Sex	Male	1			
	Female	0.73	0.63	0.86	**0.000**
Age	≤30	1			
	30–49	1.01	0.83	1.22	0.939
	50–69	1.10	0.91	1.33	0.337
	≥70	1.15	0.79	1.67	0.475
Nationality	China (East Asia)	1			
	Mongolia (East Asia)	2.01	1.39	2.90	**0.000**
	Vietnam (Southeast Asia)	1.33	1.07	1.67	**0.012**
	Thailand (Southeast Asia)	5.28	3.36	8.29	**0.000**
	Other Southeast Asia	1.32	1.04	1.68	**0.023**
	Central and Northern Asia	2.96	2.09	4.19	**0.000**
	Others	1.06	0.79	1.43	0.692
Living arrangement	With family	1			
	With non-family	1.98	1.42	2.75	**0.000**
	Live alone	2.39	1.99	2.87	**0.000**
	Others	3.52	2.26	5.47	**0.000**
Smoking status	Non-smoker	1			
	Former smoker	0.99	0.78	1.26	0.943
	Current smoker	1.07	0.89	1.28	0.474
Geographic region of residence	Seoul	1			
	Incheon	1.19	0.84	1.68	0.321
	Gyeonggi	1.13	0.93	1.38	0.204
	Chungcheong	1.02	0.76	1.37	0.907
	Jeolla	0.84	0.57	1.22	0.355
	Gyeongsang	1.19	0.94	1.49	0.141
	Gangwon & Jeju	1.31	0.86	2.00	0.210
Disease profile and health system					
TB treatment history	New	1			
	Retreatment	1.34	1.07	1.68	**0.010**
Disease site	Pulmonary	1			
	Pulmonary & Extra-pulmonary	0.81	0.68	0.97	**0.024**
Drug resistance status	Non-resistant TB	1			
	Resistant TB	3.78	2.71	5.27	**0.000**
PPM program enrollment	PPM	1			
	Non-PPM	1.46	1.26	1.70	**0.000**
Care transfer	None	1			
	≥1	7.10	4.23	11.91	**0.000**
Treatment facility type	General hospital	1			
	Hospital	1.90	1.42	2.53	**0.000**
	Public health center & clinic	1.51	1.28	1.79	**0.000**
Comorbidity					
Presence of comorbidities	None	1			
	Diabetes	1.77	1.26	2.47	**0.001**
	Cancer	1.74	1.08	2.82	**0.022**
	Others	0.94	0.70	1.27	0.690

OR, Odds Ratio; CI, Confidence interval. Significant *p*-values are shown in bold.

Multivariate analysis confirmed similar patterns, with adjusted odds highest among Thai (aOR 5.73), Mongolian (aOR 2.31), and Vietnamese (aOR 1.79) migrants (Table 2). Living alone (aOR 2.31), having drug-resistant TB (aOR 4.43), diabetes (aOR 2.29), cancer (aOR 2.50), undergoing care transfers (aOR 7.91), and receiving clinic-based treatment (aOR 1.49) were all independently associated with increased risk.

**Table 2:** Determinants of lost to follow-up among patients with TB patients who were migrants, registered, and treated from 2016 to 2018.

Variables		Adjusted OR	95% CI		*p*-value
			Lower	Upper	
Sex	Male	1			
	Female	0.83	0.70	0.98	**0.031**
Nationality	China (East Asia)	1			
	Mongolia (East Asia)	2.31	1.57	3.40	**0.000**
	Vietnam (Southeast Asia)	1.79	1.40	2.29	**0.000**
	Thailand (Southeast Asia)	5.73	3.59	9.13	**0.000**
	Other Southeast Asia	1.64	1.25	2.14	**0.000**
	Central and Northern Asia	2.85	1.97	4.11	**0.000**
	Others	1.22	0.88	1.68	0.229
Living arrangement	With family	1			
	With non-family	1.82	1.26	2.62	**0.001**
	Live alone	2.31	1.91	2.81	**0.000**
	Others	3.06	1.92	4.89	**0.000**
TB treatment history	New	1			
	Retreatment	1.27	0.99	1.63	0.059
Disease site	Pulmonary	1			
	Pulmonary & Extra-pulmonary	1.04	0.84	1.28	0.725
Drug resistance status	Non-resistant TB	1			
	Resistant TB	4.43	3.06	6.40	**0.000**
PPM program enrollment	PPM	1			
	Non-PPM	1.17	0.93	1.47	0.187
Care transfer	None	1			
	≥1	7.91	4.59	13.64	**0.000**
Treatment facility type	General hospital	1			
	Public health center & clinic	1.49	1.16	1.92	**0.002**
	Hospital	1.57	1.13	2.18	**0.008**
Presence of Comorbidities	None	1			
	Diabetes	2.29	1.60	3.29	**0.000**
	Cancer	2.50	1.49	4.19	**0.001**
	Others	1.27	0.93	1.74	0.138

aOR, adjusted Odds Ratio; CI, Confidence interval; DM, diabetes mellitus. Significant *p*-values are shown in bold.

### 3.4 Qualitative insights into treatment discontinuation

By engaging nurses directly involved in TB management under the PPM framework, three major themes emerged from the in-depth interviews regarding the barriers that contribute to LTFU among migrant patients with TB (Table 3).

**Table 3:** The three themes that emerged from in-depth interviews with PPM nurses.

Themes	Primary themes	Secondary themes
Personal factor	Educational gaps hindered adherence, as many migrants perceived TB as a “mild cold” and discontinued treatment after symptom improvement.	PPM nurses emphasized the need for multilingual public health campaigns to educate migrants on the importance of completing treatment and the risks of non-adherence, including drug resistance and public health consequences.
Structural factor	Interpreter limitations exacerbated misunderstandings, as non-medical interpreters struggled to explain drug side effects or regimen complexities.	Medical interpretation services become available by training interpreters specifically in healthcare terminology and TB-related care
	Employer roles proved critical, with 68% of migrant workers relying on employers for basic needs.	PPM nurses proposed integrating treatment monitoring into workplace systems to leverage employers’ roles in ensuring adherence.
Health service factor	Short term visitors and returnees with TB are often not informed about the appropriate transfer-out procedures.	Advocacy efforts were recommended to increase migrant awareness of their eligibility for PPM enrollment and free TB treatment, as well as transfer-out procedures.
	Migrants without insurance frequently faced hidden fees at non-PPM facilities, where they frequently failed to convey the risks of premature discontinuation	
	Data showed that 43% of LTFU cases involved patients avoiding follow-up due to unanticipated costs.	

## 4 Discussion

This study offers crucial insights into the significant challenge of TB treatment adherence among foreign migrants in ROK, revealing a substantially higher rate of LTFU compared to native Korean patients. Despite comprising only 3.5% of the total population, foreign migrants accounted for over 5% of annual TB cases between 2016 and 2018, with an average incidence rate of 178.2 per 100,000, approximately 2.5 times higher than the national average. This disproportionate burden highlights an urgent need for targeted interventions.

The follow-up discontinuation rate among migrant patients with TB was significantly higher (21.5%) than that among domestic patients (2.3%). This significant disparity poses a critical barrier to global TB elimination efforts (26, 27). Previous research indicates that migration itself increases the likelihood of treatment discontinuation, with foreign nationals showing a higher tendency to disengage from care (19, 28). The mixed-methods approach utilized in this study provided a comprehensive understanding of the various determinants contributing to this issue. Several sociodemographic factors were found to profoundly influence LTFU among migrants.

Nationality emerged as a critical determinant of LTFU, with higher discontinuation rates seen among those from Thailand, Mongolia, and Vietnam compared to Chinese nationals. These elevated rates may partially due to systemic misclassification; Many migrants leave ROK for further care without formal transfer procedures, resulting in delayed immigration verification and erroneous classification as LTFU. Previous studies have similarly found that patients who depart without notifying health staff are flagged as LTFU once their exit is confirmed (19). Migrants with repeated treatment interruptions are often prioritized for deportation once non-infectious, contributing to higher discontinuation rates compared to residents.

Living arrangement also strongly influenced follow-up adherence. Migrants in single-person or non-family households were more likely to discontinue care than those living with their families, reflecting a lack of social and emotional support, less favorable living conditions, and limited access to healthcare (29, 30). Family presence is widely recognized as a protective factor, promoting adherence through motivation and oversight for medication adherence (31). Additional barriers—such as stigma, psychological distress, and insufficient treatment support—further hinder continuity of care for individuals living alone or in non-family environments (32, 33). Integration of housing support programs with local social welfare services has demonstrated effectiveness in mitigating risks such as drug-resistant TB, relapse, and mortality, ultimately improving overall treatment outcomes (28, 34).

The analysis identified a consistent gender disparity in TB treatment adherence, with female sex associated with lower odds of LTFU in both univariable (OR 0.73) and multivariate (aOR 0.83) models. This suggests that sex-specific factors may shape adherence patterns. Migrant females were more likely to live with family (47.8%) than males (29.3%), while males predominantly lived alone (59.5%). Since family support plays a key role in promoting medication adherence through emotional and practical support, this difference in living arrangements likely contributes to the disparity. Additionally, females reported lower smoking rates (95.3% non-smokers) versus males (37.5% current, 17.7% former smokers), indicating healthier behaviors that may support treatment completion. Females were also more often enrolled in the PPM program (58.5% vs. 49.2%), enhancing their access to structured nurse-led monitoring. These overlapping factors—social support, lifestyle, and program engagement—underscore the multifaceted gendered influences on treatment outcomes.

Clinical factors also contributed to the high LTFU rates. Drug-resistant TB significantly increased the risk of treatment discontinuation, with affected patients being 4.43 times more likely to be LTFU than those with drug-susceptible TB. The extended treatment duration, often triple that of standard regimens, and the severe side effects of second-line drugs contributes to voluntary interruption (35). These challenges underscore the need for enhanced adherence strategies, including continuous monitoring, comprehensive patient education, as well as targeted support for those with drug-resistant TB (12, 36, 37). While less common among migrants than natives, the presence of comorbidities such as diabetes and cancer significantly increased the odds of LTFU for migrants. This highlights the need for integrated care that addresses co-existing health conditions, a point also recognized in the National TB Management Guidelines which list diabetes and cancer as high-risk factors for TB progression (38).

Crucial systemic and health system factors were also identified. Migrants who experienced one or more care transfers were significantly more likely to be LTFU. This indicates that frequent transfers disrupt treatment continuity due to delays in record sharing, inconsistent protocols, and insufficient communication (39). Also, migrants had less access to PPM nurses (52.9%) compared to native Koreans (73.9%). PPM nurses are crucial for patient management, including initial investigations for reported patients and conducting vulnerability assessments for customized care (19, 40). The qualitative findings revealed that this disparity was compounded by low awareness among migrants of the availability of free TB treatment under the PPM program, leading to perceived costs at non-PPM facilities and avoidance of follow-up.

PPM nurses reported that many migrants perceived TB as a “mild cold” and prematurely discontinued treatment once symptoms improved, and are unaware of the significant risks associated with incomplete care, such as the development of drug-resistant TB and the possibility of relapse (31, 41). This underscores the urgent need for culturally and linguistically tailored educational campaigns that clearly explain TB’s severity, the importance of completing the full treatment course, and the risks of drug resistance and relapse (41–43). The qualitative data also highlighted significant language barriers and limitations of non-medical interpreters in effectively explaining complex medical information, such as drug side effects and regimen complexities. Improving medical interpretation services with trained professionals possessing specialized healthcare and TB-related knowledge is crucial to bridge these communication gaps.

These findings resonate with existing literature emphasizing that patient education, robust social support, and accessible, consistent care are fundamental to improving TB treatment adherence (19, 31). The study’s insights reinforce that overcoming high LTFU rates among migrant TB patients in the ROK necessitates a multi-faceted approach that extends beyond clinical management to address underlying social, cultural, and systemic barriers. This implies the need for proactive outreach, culturally sensitive health education, strengthened interpreter services, and policy reforms to ensure seamless, affordable, and comprehensive care for this vulnerable population. The “Tuberculosis Relief Belt” initiative represents a positive step in strengthening patient referral systems and providing financial subsidies to vulnerable groups, including support for treatment costs, outsourced medical expenses, caregiving, nutrition, and patient transport (28, 38).

While this study offers valuable insights, it is important to acknowledge its limitations. The authors themselves noted the lack of detailed clinical data, such as lung X-rays or smear/culture data, which could have made the study more robust. Similarly, the absence of detailed socioeconomic indicators (e.g., visa type, specific occupation, income, education level) limited a more nuanced understanding of disease progression and socioeconomic vulnerabilities. Additionally, the small sample size of multidrug-resistant cases restricted a thorough examination of its specific association with LTFU. Future research should aim to incorporate these variables for a more complete understanding.

### 4.1 Recommendations

To counter the perception of TB as a “mild cold” and prevent premature treatment discontinuation, the 2025 National TB Management Guidelines already highlight patient counseling and education as vital for successful treatment completion, covering transmission, adherence, and side effect monitoring (31). This existing framework can be leveraged to launch targeted, multilingual public health campaigns that explicitly detail the TB treatment process, emphasize the necessity of completing the full course, and explain the significant risks of non-adherence, such as drug resistance and relapse. These campaigns should utilize accessible materials, potentially drawing from resources on the “TB ZERO website” (44). Public-Private Mix (PPM) nurses and public health center TB management personnel are ideally positioned to deliver this critical education, as their roles include patient counseling and education.

Eligible migrant patients should be actively connected to ROK’s expanded “Tuberculosis Relief Belt” initiative, which is already recognized as a “pivotal measure to close gaps in TB care.” This initiative strengthens patient referral systems and provides targeted financial subsidies for socioeconomically vulnerable populations—including support for treatment costs, outsourced medical expenses, caregiving, nutrition, and patient transport (28, 38). Migrants are identified as a socioeconomically vulnerable group. The National Medical Center is specifically tasked with operating this support program.

Access to PPM programs should be also expanded by increasing the presence of dedicated PPM nurses in primary healthcare facilities, where many migrants receive initial care but which currently “lack dedicated PPM nurses.” Simultaneously, establish clear pathways for integrated care that ensures migrants with comorbidities, such as diabetes and cancer, receive specialized management. These conditions are recognized as high-risk factors in the National TB Management Guidelines, and primary care facilities often lack the necessary specialists. PPM is one of the most prioritized flagship programs under the Korean Diseases Control Agency, where its coverage has been gradually increased up to 81.5% (45).

In conclusion, this study demonstrated that migrant patients with TB in the ROK experience significantly higher rates of LTFU than native patients. The key factors contributing to this disparity include nationality, living arrangement, gender, drug-resistant TB, the presence of comorbidities, frequent care transfers, and type of treatment facility. Migrants from certain countries are disproportionately affected and often discontinue care due to transnational movements and limited support systems. Social isolation, particularly among those living alone or in non-family households, further increases the risk of treatment discontinuation, whereas the prolonged treatment required for drug-resistant TB creates additional adherence challenges. Qualitative insights further revealed barriers, such as educational gaps, misconceptions, poor medical interpretation, and low awareness of free treatment and PPM eligibility. Addressing these issues requires culturally tailored education, employer support, improved interpretation services, and a streamlined care system.

## Data Availability

The data analyzed in this study is subject to the following licenses/restrictions: the dataset is not in public domain as it is part of the national TB surveillance data. Requests to access these datasets should be directed to Sumin Jeon, jeonsumin38@gmail.com.
