# Supplementary material for: Determinants of the lost to follow-up status among patients with tuberculosis who emigrated to the Republic of Korea: a mixed-method study
Source: Front Public Health. 2025 Sep 12;13:1641182. doi: 10.3389/fpubh.2025.1641182 (PMC12463823; doi:10.3389/fpubh.2025.1641182)
Supplement: Supplementary file 1 [file Table_1.DOCX]

**Supplementary Table S1. Characteristics of participants for qualitative assessment**

| No | Position | Gender | Affiliation (Level of care) |
| --- | --- | --- | --- |
| 1 | Specialist doctor | Man | National Hospital (Tertiary) |
| 2 | PPM nurse | Woman | National Hospital (Tertiary) |
| 3 | PPM nurse | Woman | National Hospital (Tertiary) |
| 4 | TB Relief Belt nurse | Woman | National Hospital (Tertiary) |
| 5 | PPM nurse | Man | Private University Hospital (Tertiary) |
| 6 | PPM nurse | Woman | Private Hospital (Secondary) |
| 7 | PPM nurse | Woman | Private Hospital (Secondary) |
