# Supplementary material for: Determinants of the lost to follow-up status among patients with tuberculosis who emigrated to the Republic of Korea: a mixed-method study
Source: Front Public Health. 2025 Sep 12;13:1641182. doi: 10.3389/fpubh.2025.1641182 (PMC12463823; doi:10.3389/fpubh.2025.1641182)
Supplement: Supplementary file 2 [file Table_2.DOCX]

**Supplementary Table S2.** Summary of tuberculosis (TB) diagnosis, standard treatment and monitoring, and establishment of infection control/cure procedures according to “National TB Management Guideline 2025”

| **Diagnosis** | - In the Republic of Korea, TB is a "nationally notifiable disease," and all new diagnoses must be reported to community health centers within 24 hours. - The Guidelines define active TB diagnosis as either "bacteriologically confirmed TB" (positive acid-fast bacilli smear, culture, or nucleic acid amplification test like TB-PCR or Xpert MTB/RIF) or "clinically diagnosed TB" (based on symptoms, imaging such as chest X-ray or CT, histological examination, and initiation of anti-TB drugs, even without bacteriological confirmation). - The initial TB test must be conducted and can include chest X-ray, smear, culture, nucleic acid amplification test (TB-PCR), Xpert MTB/RIF, or tissue biopsy. - Initial sputum samples require at least 2, preferably 3, for smear and culture tests, with smear results typically available within 24 hours. - These diagnostic methods apply to all patients, regardless of nationality, within the Korean public and private health services. |
| --- | --- |
| **Standard Treatment** | - •Drugs, Dose, and Time: Standard anti-TB treatment in Korea involves a combination therapy with 2HRZE/4HR(E)* for 6 months or more. - For multidrug-resistant TB (MDR-TB), treatment duration is considerably extended, often more than three times that of drug-susceptible TB, ranging from 6 to 20 months depending on the specific regimen and patient characteristics. - New drugs for MDR-TB (Bedaquiline, Delamanid, Pretomanid) are available but require prior review by a committee of TB specialists. |
| **Cure/Treatment Success Criteria** | - Treatment outcomes, including "successful" (cure or treatment completion) and "unsuccessful" (treatment failure, LTFU, or death), are defined according to 2020 World Health Organization (WHO) guidelines. - "Cure" specifically requires bacteriological confirmation at the start of treatment, completion of the regimen, and bacteriological conversion (two consecutive negative culture or smear results at least 7 days apart) with no evidence of treatment failure. |
| **Adherence monitoring** | - Adherence is monitored by Public-Private Mix (PPM) nurses, who provide education, counseling, and monitoring treatment adherence. - Directly Observed Treatment (DOT), implemented through various methods, including remote video medication confirmation (telemedicine) provided by the Korean National Tuberculosis Association (KNTTA) as part of their TB Medication Support Program. - For migrant patients and other vulnerable groups, Tuberculosis Relief Belt initiative provides pivotal measure to close gaps in TB care. This initiative strengthens patient referral systems and provides targeted financial subsidies for socioeconomically vulnerable populations—including support for treatment costs, outsourced medical expenses, caregiving, nutrition, and patient transport. |
| **Establishing Infection Control/Cure** | - The loss of infectiveness for transmissible TB patients is determined by a physician based on sputum test results (negative smear), resolution of respiratory symptoms, and completion of at least 2 weeks of effective anti-TB treatment. - Regular follow-up examinations, including clinical assessments and laboratory tests, are conducted throughout treatment to monitor progress and confirm cure/completion. |

*: **H**: Isoniazid; **R**: Rifampin; **Z**: Pyrazinamide; and **E**: Ethambutol
