# Supplementary material for: Determinants of the lost to follow-up status among patients with tuberculosis who emigrated to the Republic of Korea: a mixed-method study
Source: Front Public Health. 2025 Sep 12;13:1641182. doi: 10.3389/fpubh.2025.1641182 (PMC12463823; doi:10.3389/fpubh.2025.1641182)
Supplement: Supplementary file 3 [file Table_3.DOCX]

**Supplementary Table S3.** Characteristics of the patients with TB who were registered in the ROK from 2016 to 2018 according to their nationality

| **Variables** | | | Migrants | |  |  |  |  | *p*-value | Korean | |  |  |  |  | *p*-value |
| --- | --- | --- | --- | --- | --- | --- | --- | --- | --- | --- | --- | --- | --- | --- | --- | --- |
|  | | | Total | | Male |  | Female |  |  | Total | | Male |  | Female |  |  |
|  | | | N=4,011 | % | N=2,415 | % | N=1,596 | % |  | N=64,620 | % | N=37,876 | % | N=26,744 | % |  |
| **Yearly Distribution** | | |  |  |  |  |  |  |  |  |  |  |  |  |  |  |
| Year | 2016 | | 1,710 | 42.6 | 1,029 | 42.6 | 681 | 42.7 | 0.841 | 24,366 | 37.7 | 14,331 | 37.8 | 10,035 | 37.5 | 0.099 |
|  | 2017 | | 1,225 | 30.5 | 731 | 30.3 | 494 | 30.9 |  | 20,809 | 32.2 | 12,073 | 31.9 | 8,736 | 32.7 |  |
|  | 2018 | | 1,076 | 26.8 | 655 | 27.1 | 421 | 26.4 |  | 19,445 | 30.1 | 11,472 | 30.3 | 7,973 | 29.8 |  |
| **Sociodemographic profiles** |  | |  |  |  |  |  |  |  |  |  |  |  |  |  |  |
| Age | ≤30 | | 1,234 | 30.8 | 642 | 26.6 | 592 | 37.1 | <0.001 | 6,687 | 10.4 | 3,441 | 9.1 | 3,246 | 12.1 | <0.001 |
|  | 30-49 | | 1,375 | 34.3 | 894 | 37.0 | 481 | 30.1 |  | 15,066 | 23.3 | 8,853 | 23.4 | 6,213 | 23.2 |  |
|  | 50-69 | | 1,223 | 30.5 | 810 | 33.5 | 413 | 25.9 |  | 22,762 | 35.2 | 15,470 | 40.8 | 7,292 | 27.3 |  |
|  | ≥70 | | 179 | 4.5 | 69 | 2.9 | 110 | 6.9 |  | 20,105 | 31.1 | 10,112 | 26.7 | 9,993 | 37.4 |  |
| Nationality | China (East Asia) | | 2,295 | 57.2 | 1,429 | 59.2 | 866 | 54.3 | <0.001 |  |  |  |  |  |  |  |
|  | Mongolia (East Asia) | | 145 | 3.6 | 53 | 2.2 | 92 | 5.8 |  |  |  |  |  |  |  |  |
|  | Vietnam (Southeast Asia) | | 547 | 13.6 | 307 | 12.7 | 240 | 15.0 |  |  |  |  |  |  |  |  |
|  | Thailand (Southeast Asia) | | 83 | 2.1 | 39 | 1.6 | 44 | 2.8 |  |  |  |  |  |  |  |  |
|  | Other Southeast Asia | | 475 | 11.8 | 264 | 10.9 | 211 | 13.2 |  |  |  |  |  |  |  |  |
|  | Central and Northern Asia | 149 | | 3.7 | 90 | 3.7 | 59 | 3.7 |  |  |  |  |  |  |  |  |
|  | Others | 317 | | 7.9 | 233 | 9.7 | 84 | 5.3 |  |  |  |  |  |  |  |  |
| Living arrangement | With family | 1,470 | | 36.7 | 708 | 29.3 | 762 | 47.8 | <0.001 | 33,611 | 52.0 | 19,454 | 51.4 | 14,157 | 52.9 | <0.001 |
|  | With non-family | 257 | | 6.4 | 204 | 8.4 | 53 | 3.3 |  | 234 | 0.4 | 145 | 0.4 | 89 | 0.3 |  |
|  | Live alone | 2,183 | | 54.4 | 1,436 | 59.5 | 747 | 46.8 |  | 29,103 | 45.0 | 17,360 | 45.8 | 11,743 | 43.9 |  |
|  | Others | 101 | | 2.5 | 67 | 2.8 | 34 | 2.1 |  | 1,672 | 2.6 | 917 | 2.4 | 755 | 2.8 |  |
| Smoking status | Non-smoker | 2,603 | | 64.9 | 1,082 | 44.8 | 1,521 | 95.3 | <0.001 | 40,610 | 62.8 | 15,492 | 40.9 | 25,118 | 93.9 | <0.001 |
|  | Former smoker | 459 | | 11.4 | 428 | 17.7 | 31 | 1.9 |  | 11,781 | 18.2 | 11,130 | 29.4 | 651 | 2.4 |  |
|  | Current smoker | 949 | | 23.7 | 905 | 37.5 | 44 | 2.8 |  | 12,229 | 18.9 | 11,254 | 29.7 | 975 | 3.7 |  |
| Geographic region of residence | Seoul | 1,261 | | 31.4 | 716 | 29.7 | 545 | 34.2 | 0.012 | 17,174 | 26.6 | 10,230 | 27.0 | 6,944 | 26.0 | 0.013 |
|  | Incheon | 216 | | 5.4 | 135 | 5.6 | 81 | 5.1 |  | 3,370 | 5.2 | 1,983 | 5.2 | 1,387 | 5.2 |  |
|  | Gyeonggi | 1,200 | | 29.9 | 735 | 30.4 | 465 | 29.1 |  | 12,142 | 18.8 | 7,180 | 19.0 | 4,962 | 18.6 |  |
|  | Chungcheong | 351 | | 8.8 | 222 | 9.2 | 129 | 8.1 |  | 5,715 | 8.8 | 3,314 | 8.8 | 2,401 | 9.0 |  |
|  | Jeolla | 211 | | 5.3 | 116 | 4.8 | 95 | 6.0 |  | 6,020 | 9.3 | 3,506 | 9.3 | 2,514 | 9.4 |  |
|  | Gyeongdang | 644 | | 16.1 | 416 | 17.2 | 228 | 14.3 |  | 16,817 | 26.0 | 9,689 | 25.6 | 7,128 | 26.7 |  |
|  | Gangwon & Jeju | 128 | | 3.2 | 75 | 3.1 | 53 | 3.3 |  | 3,382 | 5.2 | 1,974 | 5.2 | 1,408 | 5.3 |  |
| **Disease profiles and health system** | |  | |  |  |  |  |  |  |  |  |  |  |  |  |  |
| TB treatment history | New | 3,565 | | 88.9 | 2,139 | 88.6 | 1,426 | 89.3 | 0.444 | 55,670 | 86.2 | 31,664 | 83.6 | 24,006 | 89.8 | <0.001 |
|  | Retreatment | 446 | | 11.1 | 276 | 11.4 | 170 | 10.7 |  | 8,950 | 13.9 | 6,212 | 16.4 | 2,738 | 10.2 |  |
| Disease site | Pulmonary | 3,004 | | 74.9 | 1,870 | 77.4 | 1,134 | 71.0 | <0.001 | 46,360 | 71.7 | 28,290 | 74.7 | 18,070 | 67.6 | <0.001 |
|  | Pulmonary & Extra-pulmonary | 1,007 | | 25.1 | 545 | 22.6 | 462 | 29.0 |  | 18,260 | 28.3 | 9,586 | 25.3 | 8,674 | 32.4 |  |
| Drug resistance status | Non-resistant TB | 3,858 | | 96.2 | 2,307 | 95.5 | 1,551 | 97.2 | 0.007 | 63,682 | 98.6 | 37,278 | 98.4 | 26,404 | 98.7 | 0.001 |
|  | Resistant TB | 153 | | 3.8 | 108 | 4.5 | 45 | 2.8 |  | 938 | 1.5 | 598 | 1.6 | 340 | 1.3 |  |
| PPM program enrollment | Non-PPM | 1,889 | | 47.1 | 1,226 | 50.8 | 663 | 41.5 | <0.001 | 16,901 | 26.2 | 10,317 | 27.2 | 6,584 | 24.6 | <0.001 |
|  | PPM | 2,122 | | 52.9 | 1,189 | 49.2 | 933 | 58.5 |  | 47,719 | 73.9 | 27,559 | 72.8 | 20,160 | 75.4 |  |
| Care Transfer | None | 3,943 | | 98.3 | 2,369 | 98.1 | 1,574 | 98.6 | 0.206 | 63,956 | 99.0 | 37,406 | 98.8 | 26,550 | 99.3 | <0.001 |
|  | ≥1 | 68 | | 1.7 | 46 | 1.9 | 22 | 1.4 |  | 664 | 1.0 | 470 | 1.2 | 194 | 0.7 |  |
| Treatment facility type | Public health center and clinic | 1,112 | | 27.7 | 752 | 31.1 | 360 | 22.6 | <0.001 | 3,887 | 6.0 | 2,544 | 6.7 | 1,343 | 5.0 | <0.001 |
|  | Hospital | 247 | | 6.2 | 151 | 6.3 | 96 | 6.0 |  | 3,966 | 6.1 | 2,409 | 6.4 | 1,557 | 5.8 |  |
|  | General hospital | 2,652 | | 66.1 | 1,512 | 62.6 | 1,140 | 71.4 |  | 56,767 | 87.9 | 32,923 | 86.9 | 23,844 | 89.2 |  |
| **Comorbidity** |  |  | |  |  |  |  |  |  |  |  |  |  |  |  |  |
| Presence of comorbidities | None | 3,467 | | 86.4 | 2,104 | 87.1 | 1,363 | 85.4 | <0.001 | 35,626 | 55.1 | 20,463 | 54.0 | 15,163 | 56.7 | <0.001 |
|  | Diabetes | 168 | | 4.2 | 124 | 5.1 | 44 | 2.8 |  | 9,108 | 14.1 | 6,060 | 6.0 | 3,048 | 11.4 |  |
|  | Cancer | 79 | | 2.0 | 45 | 1.9 | 34 | 2.1 |  | 4,116 | 6.4 | 2,682 | 7.1 | 1,434 | 5.4 |  |
|  | Others | 297 | | 7.4 | 142 | 5.9 | 155 | 9.7 |  | 15,770 | 24.4 | 8,671 | 22.9 | 7,099 | 26.5 |  |
| **Treatment outcome** |  |  | |  |  |  |  |  |  |  |  |  |  |  |  |  |
| Treatment outcome | Success | 3,137 | | 78.2 | 1,841 | 76.2 | 1,296 | 81.2 | <0.001 | 61,634 | 95.4 | 35,896 | 94.8 | 25,738 | 96.2 | <0.001 |
|  | - Cured | 874 | | 21.8 | 557 | 23.1 | 317 | 19.9 |  | 12,974 | 20.1 | 8,115 | 21.4 | 4,859 | 18.2 |  |
|  | - Completed | 2,263 | | 56.4 | 1,284 | 53.2 | 979 | 61.3 |  | 48,660 | 75.3 | 27,781 | 73.4 | 20,879 | 78.1 |  |
|  | Fail | 874 | | 21.8 | 574 | 23.8 | 300 | 18.8 |  | 2,986 | 4.6 | 1,980 | 5.2 | 1,006 | 3.8 |  |
|  | - Failed | 2 | | 0.1 | 1 | 0.0 | 1 | 0.1 |  | 31 | 0.1 | 28 | 0.1 | 3 | 0.0 |  |
|  | - Lost to follow-up | 863 | | 21.5 | 569 | 23.6 | 294 | 18.4 |  | 1,455 | 2.3 | 987 | 2.6 | 468 | 1.8 |  |
|  | - Died | 9 | | 0.2 | 4 | 0.2 | 5 | 0.3 |  | 1,500 | 2.3 | 965 | 2.6 | 535 | 2.0 |  |
